# PCOSBase: a manually curated database of polycystic ovarian syndrome

**DOI:** 10.1093/database/bax098

**Published:** 2017-12-19

**Authors:** Nor Afiqah-Aleng, Sarahani Harun, Mohd Rusman Arief A-Rahman, Nor Azlan Nor Muhammad, Zeti-Azura Mohamed-Hussein

**Affiliations:** 1 Institute of Systems Biology (INBIOSIS); 2 Pusat Pengajian Biosains dan Bioteknologi, Fakulti Sains dan Teknologi, Universiti Kebangsaan Malaysia, 43600 UKM Bangi, Selangor, Malaysia

## Abstract

Polycystic ovarian syndrome (PCOS) is one of the main causes of infertility and affects 5–20% women of reproductive age. Despite the increased prevalence of PCOS, the mechanisms involved in its pathogenesis and pathophysiology remains unclear. The expansion of omics on studying the mechanisms of PCOS has lead into vast amounts of proteins related to PCOS resulting to a challenge in collating and depositing this deluge of data into one place. A knowledge-based repository named as PCOSBase was developed to systematically store all proteins related to PCOS. These proteins were compiled from various online databases and published expression studies. Rigorous criteria were developed to identify those that were highly related to PCOS. They were manually curated and analysed to provide additional information on gene ontologies, pathways, domains, tissue localizations and diseases that associate with PCOS. Other proteins that might interact with PCOS-related proteins identified from this study were also included. Currently, 8185 PCOS-related proteins were identified and assigned to 13 237 gene ontology vocabulary, 1004 pathways, 7936 domains, 29 disease classes, 1928 diseases, 91 tissues and 320 472 interactions. All publications related to PCOS are also indexed in PCOSBase. Data entries are searchable in the main page, search, browse and datasets tabs. Protein advanced search is provided to search for specific proteins. To date, PCOSBase has the largest collection of PCOS-related proteins. PCOSBase aims to become a self-contained database that can be used to further understand the PCOS pathogenesis and towards the identification of potential PCOS biomarkers.

**Database URL**: http://pcosbase.org

## Introduction

Polycystic ovarian syndrome (PCOS) is an endocrine disorder that is characterized by a combination of two out of three features, i.e. ovulatory dysfunction, hyperandrogenism and/or the presence of polycystic ovaries (1). PCOS is difficult to diagnose as these features might lead to various phenotypic manifestations (2). Clinical findings showed that women with PCOS have higher risk to develop other complications such as endometrial cancer (3), diabetes (4), hypertension (5) and depression (6). These phenotypic manifestations and disease associations would significantly interrupt the progress in deciphering the cause of PCOS (7).

Transcriptomics (8) and proteomics (9) were used to identify genes and proteins differences between non-PCOS and PCOS women and the resulting data analysis could be used to elucidate the cause of PCOS. At present, numbers of published expression studies has increased significantly since 2003, and this contributes to the vast amount of PCOS-related molecular data. Unfortunately, these molecular data were randomly distributed in various general biological databases (GenBank and UniProt) and literatures thus contribute to the difficulties in finding all genes and proteins that are related to PCOS. This limitation has led us to develop PCOSBase to house 8185 PCOS-related proteins that were manually curated. These proteins were filtered from 17 492 identified proteins from 30 expression studies and 9 databases. Bioinformatic analyses were performed on these proteins to characterize and classify them into specific datasets based on their molecular characteristics. PCOSBase also provides indexed publications related to PCOS. These features signify the differences of PCOSBase to previously published, PCOSKB (10) (PCOSKB statistics as of July 2017 contains 241 sequences). Detailed information on proteins and diseases related to PCOS can be found in PCOSBase but none on the proteins-drugs association as described in Open Targets (www.targetvalidation.org). Open Targets has listed 1119 proteins identified as drug targets for PCOS and 73% of those can be found in PCOSBase (11). PCOS is a focus in this study due to inadequate information and understanding on its complex molecular mechanism and at the same time it associates with many well-described diseases identified from clinical findings. For this reason, PCOSBase serves as a comprehensive medically oriented repository that will be an excellent aid in providing and integrating accurate molecular information for in depth understanding on PCOS.

Herein, the development and current status of PCOSBase were described. The provided web interfaces were further systematically discussed. PCOSBase can be accessed online at http://pcosbase.org (PCOSBase v1.0, last updated on 21 November 2017).

## Materials and methods

### Data collection

Keywords including ‘Polycystic Ovary Syndrome,’ ‘Polycystic Ovary Syndrome 1,’ ‘PCOS,’ ‘polycystic ovaries,’ ‘PCOS,’ ‘PCO,’ ‘PCO1,’ ‘Stein-Leventhal,’ ‘Stein Leventhal,’ ‘Stein-Leventhal Syndrome,’ ‘Polycystic Ovary Disease,’ ‘Polycystic Ovarian Disease,’ ‘PCOD,’ ‘Sclerocystic Ovarian Degeneration,’ ‘Sclerocystic Ovary Syndrome,’ ‘Sclerocystic Ovarian Disease’ and ‘Bilateral PCOS’ were searched in nine disease-associated databases including OMIM (12), HGMD (13), DisGeNET (14), MalaCards (15), PhenomicDb (16), DISEASES (17), DGA (18), GWASdb (19) and GWAS catalog (20).

Previous keywords of PCOS and another keywords such as ‘gene expression,’ ‘protein expression,’ ‘expression,’ ‘transcriptomics,’ ‘proteomics’ or ‘microarray’ were also used to search for relevant publications from PubMed (21), ArrayExpress (22), ScienceDirect and Scopus. Genes and proteins that were significantly expressed in those publications were included as PCOS-related proteins. These publications were indexed and listed in PCOSBase.

All genes and proteins from disease-associated databases and published expression publications were compared against NCBI Gene (23) and UniProt (24) databases to obtain their unique Gene ID and UniProt ID. The overlapping data that were obtained in more than one database or studies were combined.

### Functional annotations

To better understand the function of PCOS-related proteins, extensive information on the proteins such as chromosomal location, gene ontology (GO), pathway, proteins structural information, tissue localization, disease-related information and protein-protein interaction (PPI) were retrieved from online databases such as NCBI Gene (23), UniProt (24), Gene Ontology Consortium (25), KEGG (26), BioCarta (27), WikiPathways (28), Interpro (29), Human Protein Atlas (30), DisGeNET (14) and HIPPIE (31), or were obtained from our bioinformatics analysis (where necessary).

### Database organization and architecture

**Figure 1.**
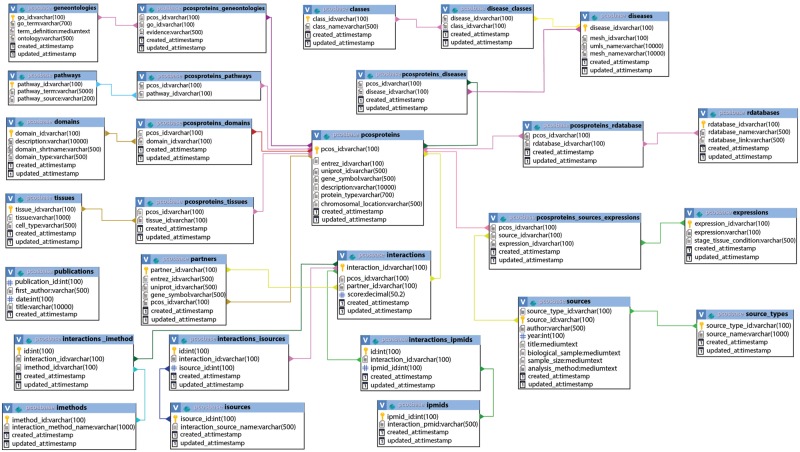
PCOSBase schema. This schema shows all the 29 tables with the connections from table to table.

All collected data including relevant information on PCOS-related proteins, functional annotation information and PCOS publications were organized in 29 tables. The 28 tables were linked to each other except for PCOS publications table (Figure 1).

PCOSBase was built as a relational database using MySQL Server 5.0.11. The web interfaces were designed using Laravel 5.4 (PHP web framework), HTML and JavaScript.

## Results and Discussion

### Database summary

**Figure 2.**
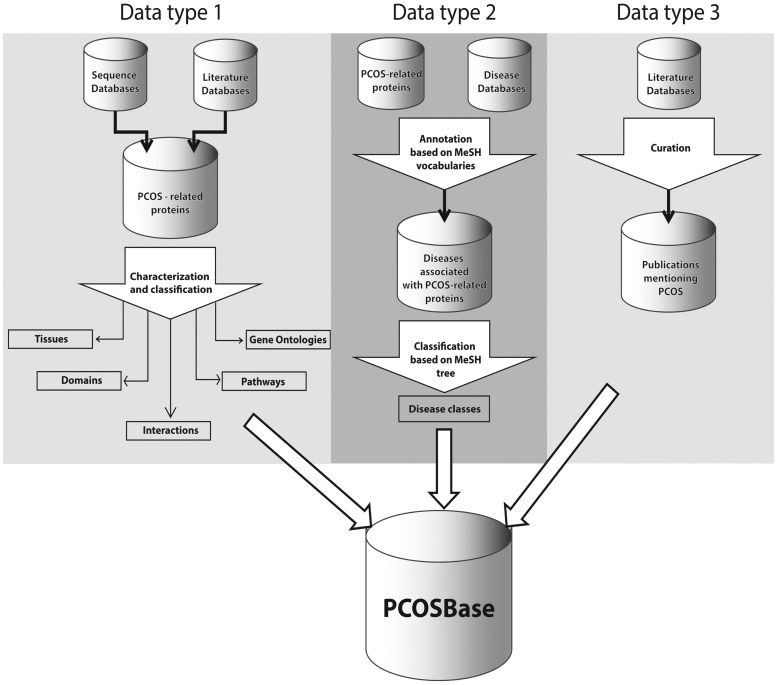
PCOSBase data types structure organization. These data types are tables that can be found in Browse and Datasets menu.

Figure 2 depicts the organization of three data types in PCOSBase; i.e. PCOS-related proteins, diseases and publications. Currently, PCOSBase contains 8185 PCOS-related proteins retrieved from nine databases and 30 expression studies. Characterization on these proteins have resulted to the classification into 13 237 GOs, 7936 domains, 91 tissues with cell types, 320 472 interactions and 1004 pathways where most of the proteins are located in the metabolic pathways. Prediction on the diseases associated to PCOS reveals 1928 diseases. These were classified into 29 disease classes. Publications of 14 368 articles on PCOS are indexed in this database. Numbers of entries in each dataset were summarized in Table 1.

**Table 1.** Number of entries in the datasets of PCOSBase

Dataset	Entries
PCOS-related proteins	8185
Gene ontologies	13 237
Biological processes	8971
Cellular components	1305
Molecular functions	2961
Domains	7936
Pathways	1004
Interactions	320 472
PCOS-related diseases	1928
Disease classes	29
Tissues	91
Databases	9
Resources	30
Transcriptomics	19
Proteomics	11
Publications	14 368

### Database interface and access

PCOSBase interface contains six main menus, i.e. About, Search, Browse, Datasets, Network and Help that will help the user to easily navigate the respective pages.

**Figure 3.**
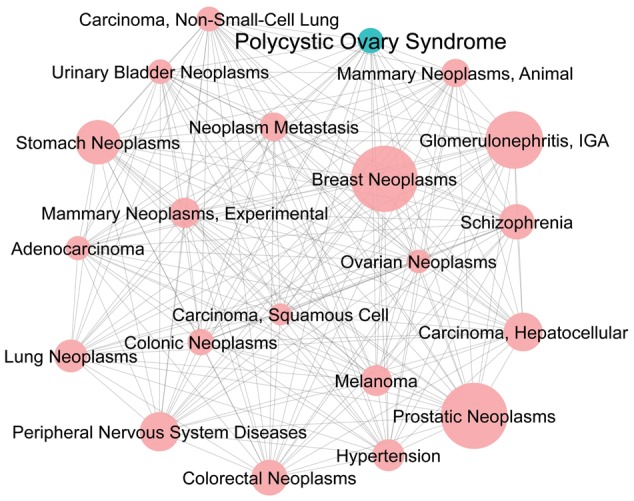
PCOS-disease interaction network. This network is predicted based on PPI and 20 diseases have been predicted to be highly associated with PCOS. The network demonstrates the complexity of PCOS-diseases association and the size of the nodes indicates the degree of association between PCOS and diseases. Green node represents PCOS and size of each node denotes number of shared proteins between PCOS and its respective associated disease.

- Homepage displays total data statistics in every table and five menus that will navigate the users to the pages as described below. Search box is also provided on this page.
- Information on PCOSBase and PCOS can be accessed on ‘About page.’
- ‘Search page’ provides two search options, i.e. Simple Search and Protein Advanced Search. The function of Simple Search is similar to the Search box on the homepage. Users can search for protein, GO, pathway, disease, domain and tissue that match to a particular keyword. For example, if ‘androgen’ keyword is searched, all entries in PCOSBase that contain ‘androgen’ term will appear. However, Protein Advanced Search allows the users to retrieve information of protein(s) with a particular combination of annotation. For instance, protein(s) associated with both GO term of ‘single fertilization’ and disease of ‘female infertility.’ Protein Advanced Search gives the users an option to find protein(s) that contain any combination from six different fields (protein description, GO, pathway, domain, tissue and disease).
- Users can assess all 11 datasets in PCOSBase by ‘Browse page.’ These datasets were classified based on their biological information, as described below:
  - PCOS-related proteins dataset: contains lists of 8185 proteins related to PCOS that were retrieved from various sources.
  - GO dataset: contains GO vocabulary information on all PCOS-related proteins.
  - Pathways dataset: contains all identified pathways where PCOS-related proteins are involved in.
  - Interactions dataset: contains information on PPIs of PCOS-related proteins.
  - Domains dataset: contains information on the domains present in all PCOS-related proteins.
  - Tissues dataset: provides information on which tissues and cell types where PCOS-related proteins were expressed.
  - Databases dataset: contains list of publicly available databases, where PCOS-related proteins were obtained.
  - Resources dataset: contains the expression studies of all PCOS-related proteins retrieved from transcriptomic and proteomic data.
  - PCOS-related diseases dataset: contains identified diseases that are related to PCOS-related proteins.
  - Disease classes dataset: contains information on PCOS-related diseases based on Medical Subject Headings tree.
  - Publications dataset: provides all publications from PubMed that relates to PCOS.
- Datasets dropdown menu links all datasets in PCOSBase. Datasets tab are placed at the header and appear on every page of PCOSBase, which allow the users to quickly select and redirect to their desired datasets page.
- Network menu contains all networks constructed using PCOS-related proteins, Interactions and PCOS-related diseases datasets. Currently, PCOSBase only provides several static PCOS networks. Figure 3 is one of the networks that can be found in this menu, where this network clearly depicted the association of PCOS with other diseases.
- Help menu provides the user manual of PCOSBase, database schema and all the references that were used to retrieve the data. All terms, definition and references that were used in PCOSBase were also provided in the Help page.

Each entry in PCOSBase provides brief description. For example, if the user searches or selects one of the proteins in PCOSBase, for instance ‘androgen receptor,’ it will navigate the user to the Description page of ‘androgen receptor.’ Seven tabs containing different information of ‘androgen receptor’ will appear. If the user clicks on one of the entries in GO tab, it will redirect to the description’s page of that GO. The list of PCOS-related proteins that are associated with this ontology will also appear below the GO description. The description of pathways, domains, diseases, tissues, databases, resources and partners will appear if the user clicks on those entries.

## Conclusion and future perspective 

In the next few years, the size of PCOS molecular data is expected to increase, especially with the application of new sequencing technologies such as next-generation sequencing in analysing in PCOS samples. To ensure PCOSBase is always up-to-date, all information in this database will be periodically updated. It is very important to consider a comprehensive cataloging on all types of data in any PCOS publications so as to ensure they are accessible to PCOS researchers and clinicians for their quick and easy reference. Ultimately, genomic and molecular information in this database will serve as a reliable repository that can be used to search for potential PCOS biomarker towards the development of improved diagnostics and treatment for PCOS.

## References

[1] The Rotterdam ESHRE/ASRM-Sponsored PCOS Consensus Workshop. (2004) Revised 2003 consensus on diagnostic criteria and long-term health risks related to polycystic ovary syndrome. Fertil. Steril., 81, 19–25. 10.1016/j.fertnstert.2003.10.004 14711538

[2] De Leo V., Musacchio M.C., Cappelli V. (2016) Genetic, hormonal and metabolic aspects of PCOS: an update. Reprod. Biol. Endocrinol., 14, 38. 27423183 10.1186/s12958-016-0173-x PMC4947298

[3] Li X., Shao R. (2014) PCOS and obesity : insulin resistance might be a common etiology for the development of type I endometrial carcinoma. Am. J. Cancer Res., 4, 73–79. 24482740 PMC3902234

[4] Gambineri A., Patton L., Altieri P. (2012) Polycystic ovary syndrome is a risk factor for type 2 diabetes: results from a long-term prospective study. Diabetes, 61, 2369–2374. 10.2337/db11-1360 22698921 PMC3425413

[5] Bentley-Lewis R., Seely E., Dunaif A. (2011) Ovarian hypertension: polycystic ovary syndrome. Endocrinol. Metab. Clin. North Am., 40, 433–449. 21565677 10.1016/j.ecl.2011.01.009 PMC3253555

[6] Blay S.L., Aguiar J.V.A., Passos I.C. (2016) Polycystic ovary syndrome and mental disorders: a systematic review and exploratory meta-analysis. Neuropsychiatr. Dis. Treat, 12, 2895–2903. 27877043 10.2147/NDT.S91700 PMC5108561

[7] Azziz R., Carmina E., Chen Z. (2016) Polycystic ovary syndrome. Nat. Rev. Dis. Prim., 2, 16057. 27510637 10.1038/nrdp.2016.57

[8] Lan C.-W., Chen M.-J., Tai K.-Y. (2015) Functional microarray analysis of differentially expressed genes in granulosa cells from women with polycystic ovary syndrome related to MAPK/ERK signaling. Sci. Rep., 5, 14994. 26459919 10.1038/srep14994 PMC4602237

[9] Ambekar A.S., Kelkar D.S., Pinto S.M. (2015) Proteomics of follicular fluid from women with polycystic ovary syndrome suggests molecular defects in follicular development. J. Clin. Endocrinol. Metab., 100, 744–753. 25393639 10.1210/jc.2014-2086 PMC5393508

[10] Joseph S., Barai R.S., Bhujbalrao R. (2016) PCOSKB: a knowledgebase on genes, diseases, ontology terms and biochemical pathways associated with polycystic ovary syndrome. Nucleic Acids Res., 44, D1032–D1035. 26578565 10.1093/nar/gkv1146 PMC4702829

[11] Koscielny G., An P., Carvalho-Silva D. (2017) Open targets: a platform for therapeutic target identification and validation. Nucleic Acids Res., 45, D985–D994. 27899665 10.1093/nar/gkw1055 PMC5210543

[12] Amberger J.S., Bocchini C.A., Schiettecatte F. (2015) OMIM.org: Online Mendelian Inheritance in Man (OMIM^®^), an Online catalog of human genes and genetic disorders. Nucleic Acids Res., 43, D789–D798. 25428349 10.1093/nar/gku1205 PMC4383985

[13] Stenson P.D., Mort M., Ball E.V. (2014) The Human Gene Mutation Database: building a comprehensive mutation repository for clinical and molecular genetics, diagnostic testing and personalized genomic medicine. Hum. Genet., 133, 1–9. 24077912 10.1007/s00439-013-1358-4 PMC3898141

[14] Piñero J., Bravo A., Queralt-Rosinach N. (2017) DisGeNET: a comprehensive platform integrating information on human disease-associated genes and variants. Nucleic Acids Res., 45, D833–D839. 27924018 10.1093/nar/gkw943 PMC5210640

[15] Rappaport N., Twik M., Plaschkes I. (2017) MalaCards: an amalgamated human disease compendium with diverse clinical and genetic annotation and structured search. Nucleic Acids Res., 45, D877–D887. 27899610 10.1093/nar/gkw1012 PMC5210521

[16] Kahraman A., Avramov A., Nashev L.G. (2005) PhenomicDB: a multi-species genotype/phenotype database for comparative phenomics. Bioinformatics, 21, 418–420. 10.1093/bioinformatics/bti010 15374875

[17] Pletscher-Frankild S., Pallejà A., Tsafou K. (2015) DISEASES: text mining and data integration of disease-gene associations. Methods, 74, 83–89. 25484339 10.1016/j.ymeth.2014.11.020

[18] Peng K., Xu W., Zheng J. (2013) The disease and gene annotations (DGA): an annotation resource for human disease. Nucleic Acids Res., 41, D553–D560. 23197658 10.1093/nar/gks1244 PMC3531051

[19] Li M.J., Liu Z., Wang P. (2016) GWASdb v2: an update database for human genetic variants identified by genome-wide association studies. Nucleic Acids Res., 44, D869–D876. 26615194 10.1093/nar/gkv1317 PMC4702921

[20] Welter D., MacArthur J., Morales J. (2014) The NHGRI GWAS Catalog, a curated resource of SNP-trait associations. Nucleic Acids Res., 42, D1001–D1006. 24316577 10.1093/nar/gkt1229 PMC3965119

[21] NCBI Resource Coordinators. (2017) Database resources of the National center for biotechnology information. Nucleic Acids Res., 45, D12–D17. 27899561 10.1093/nar/gkw1071 PMC5210554

[22] Kolesnikov N., Hastings E., Keays M. (2015) ArrayExpress update-simplifying data submissions. Nucleic Acids Res., 43, D1113–D1116. 25361974 10.1093/nar/gku1057 PMC4383899

[23] Brown G.R., Hem V., Katz K.S. (2015) Gene: a gene-centered information resource at NCBI. Nucleic Acids Res., 43, D36–D42. 25355515 10.1093/nar/gku1055 PMC4383897

[24] Bateman A., Martin M.J., O’donovan C. (2015) UniProt: a hub for protein information. Nucleic Acids Res., 43, D204–D212. 25348405 10.1093/nar/gku989 PMC4384041

[25] Gene Ontology Consortium. (2015) Gene ontology consortium: going forward. Nucleic Acids Res., 43, D1049–D1056. 10.1093/nar/gku1179 25428369 PMC4383973

[26] Kanehisa M., Sato Y., Kawashima M. (2016) KEGG as a reference resource for gene and protein annotation. Nucleic Acids Res., 44, D457–D462. 26476454 10.1093/nar/gkv1070 PMC4702792

[27] Nishimura D. (2001) A view from the Web, BioCarta. Biotech. Software Internet Rep., 2, 117–120.

[28] Pico A.R., Kelder T., Van Iersel M.P. (2008) WikiPathways: pathway editing for the people. PLoS Biol., 6, e184. 18651794 10.1371/journal.pbio.0060184 PMC2475545

[29] Finn R.D., Attwood T.K., Babbitt P.C. (2017) InterPro in 2017-beyond protein family and domain annotations. Nucleic Acids Res., 45, D190–D199. 27899635 10.1093/nar/gkw1107 PMC5210578

[30] Uhlén M., Fagerberg L., Hallström B.M. (2015) Tissue-based map of the human proteome. Science, 347, 1260419. 25613900 10.1126/science.1260419

[31] Alanis-Lobato G., Andrade-Navarro M.A., Schaefer M.H. (2017) HIPPIE v2.0: Enhancing meaningfulness and reliability of protein-protein interaction networks. Nucleic Acids Res., 45, D408–D414. 27794551 10.1093/nar/gkw985 PMC5210659

